# Double Attention: An Optimization Method for the Self-Attention Mechanism Based on Human Attention

**DOI:** 10.3390/biomimetics10010034

**Published:** 2025-01-08

**Authors:** Zeyu Zhang, Bin Li, Chenyang Yan, Kengo Furuichi, Yuki Todo

**Affiliations:** 1 Division of Electrical Engineering and Computer Science, Kanazawa University, Kanazawa 9201192, Japan; zeyuzhang@stu.kanazawa-u.ac.jp (Z.Z.); crislee@stu.kanazawa-u.ac.jp (B.L.); yy772013879@gmail.com (C.Y.); 2 Department of Nephrology, Kanazawa Medical University, Kahoku 9200293, Japan; furuichi@kanazawa-med.ac.jp; 3 Faculty of Electrical, Information and Communication Engineering, Kanazawa University, Kanazawa 9201192, Japan

**Keywords:** self-attention, human attention, deep learning, shifted window, medical image

## Abstract

Artificial intelligence, with its remarkable adaptability, has gradually integrated into daily life. The emergence of the self-attention mechanism has propelled the Transformer architecture into diverse fields, including a role as an efficient and precise diagnostic and predictive tool in medicine. To enhance accuracy, we propose the Double-Attention (DA) method, which improves the neural network’s biomimetic performance of human attention. By incorporating matrices generated from shifted images into the self-attention mechanism, the network gains the ability to preemptively acquire information from surrounding regions. Experimental results demonstrate the superior performance of our approaches across various benchmark datasets, validating their effectiveness. Furthermore, the method was applied to patient kidney datasets collected from hospitals for diabetes diagnosis, where they achieved high accuracy with significantly reduced computational demands. This advancement showcases the potential of our methods in the field of biomimetics, aligning well with the goals of developing innovative bioinspired diagnostic tools.

## 1. Introduction

Since the concept of the Transformer has been applied in fields from Natural Language Processing (NLP) to computer vision (CV), the efficiency and accuracy of various visual tasks have been greatly improved [1]. The major breakthrough of the Transformer is attributed to the involvement of the self-attention mechanism. The multi-head self-attention mechanism not only takes the correlation strength between each pixel and its surrounding pixels into consideration but also integrates different information learned from the multiple heads.

Inspired by human attention mechanisms, the self-attention mechanism mimics the ability of humans to focus selectively on important parts of their visual field while ignoring irrelevant details. This biologically inspired design allows Transformers to capture global dependencies in data efficiently and adaptively. The self-attention mechanism considers the global correlation strength, but when the size of the image becomes greater, it spends many learning resources on computing the correlation of each pixel with the remaining pixels [2]. Human visual systems, in contrast, allocate resources dynamically, focusing on regions of interest rather than processing every detail equally. Similarly, in Transformers, because most pixels only have relations with surrounding pixels rather than relations with those pixels that are far away, there are many meaningless calculations during the computational process of self-attention [3,4,5].

After self-attention was employed in the Vision Transformer (ViT), the Swin Transformer (Swin) was proposed to address the issue of excessive computational cost in self-attention [1,6]. The “shifted window” concept was introduced into the design of the Transformer structure to avoid unnecessary calculations, mirroring the dynamic allocation of resources observed in human vision where attention shifts spatially across the visual field, and greatly improved computational efficiency.

In the structure of self-attention, the Query, Key, and Value matrices are parameters extracted from the same image, and the computational process to obtain the three matrices represents the assessment of inter-pixel correlations [2]. The converging results of parameter matrices could reflect the importance of each pixel in the image. Through such calculations, representative image features can be obtained and the network’s complexity can be effectively reduced. Nevertheless, since the sizes of these three matrices are the same as the image, the dataset size determines the time cost in the training process [4]. Furthermore, self-attention calculations involve two matrix multiplications, and the computational cost exponentially increases with the size of the image. In the era of big data, such exponential growth poses a significant challenge. Training the entire network requires a significant amount of time and number of hardware resources, and such training often takes weeks to complete.

In human visual cognition, attention mechanisms balance local and global focus to optimize resource allocation. Inspired by this, recent approaches in neural network design have sought to emulate this dual focus to improve computational efficiency and accuracy. EfficientNet, which has made significant progress in improving convolutional neural networks and achieving high accuracy, provides a theoretical foundation for discussing the scaling of neural network width and depth [7]. In this paper, we aim to find a solution that combines self-attention with human attention to achieve better results.

The parameter matrices of the self-attention mechanism are directly generated from the image, enabling excellent performance in various visual sub-tasks. As a result, there is no need to adjust the core structure of the network when adapting it to different applications, allowing more focus on fine-tuning and improvements. Among various improvements targeting self-attention, enhancing computational efficiency is crucial. Some researchers incorporate human cognition and experience to select relatively important tokens for local attention computation [8,9]. They also employ different selection methods within each attention head to improve generalization. Zaheer’s team introduced a random selection method for token selection, further improving computational efficiency [10,11]. Also, some researchers apply clustering algorithms to cluster the Query and Key matrices and only compute the parts with matching clusters to enhance both selection stability and computational efficiency [12,13]. Wang’s team discovered that parameter matrices are often not full rank, allowing for the selection of representative values from the Key matrix to reduce computational cost [14,15]. In general, the methods for reducing the Key matrix can be broadly classified into two categories: convolutional operations on the matrix and multiplication with a mapping matrix [16,17,18]. Li’s team presented Omni-Dimensional Dynamic Convolution (ODConv), which leverages a novel attention mechanism [19,20,21]. This mechanism was inspired by the human brain’s dynamic modulation of sensory inputs, allowing it to adaptively focus on multi-dimensional features for improved efficiency and accuracy. The research on TinySaver, a dynamic model compression technique that uses independent tiny models to replace large models, demonstrated that selective attention principles in human cognition can reduce compute operations by up to 90 percent with minimal performance loss [22]. This approach mirrors how the human brain efficiently prioritizes essential information.

Refinements, including architectural adjustments, block redesign, and improved training recipes, were incorporated into DenseNets. These adjustments took inspiration from biological networks, particularly the hierarchical organization of neural circuits in the brain. The enhancements involved widening the networks and improving memory efficiency while retaining the concatenation shortcuts [23]. Astroformer employs a Transformer–convolutional hybrid with a new stack design for the network, a novel method of creating a relative self-attention layer, and pairs it with a careful selection of data augmentation and regularization techniques [24]. This hybrid design reflects the integration of localized and global attention seen in the human visual system. A gated axial-attention model was proposed, extending existing architectures by introducing an additional control mechanism in the self-attention module [25]. This mechanism mimics the gating functions in biological neurons, which regulate information flow and enhance processing efficiency.

One significant improvement of the Swin Transformer compared to the Vision Transformer is the use of the “shifted window” mechanism, which addresses the issue of information exchange between different windows. This technique parallels the brain’s ability to shift focus spatially to integrate information across regions of the visual field. In terms of improvements targeting the shifted window, CCNet computes the information of pixels along the cross path, enabling more efficient self-attention calculation [26,27]. In 2021, the SwinIR was proposed [28,29], which is composed of shallow feature extraction, deep feature extraction, and high-quality image reconstruction modules. It mimics multi-level feature processing in the human visual system, where different layers extract distinct levels of information. These modules extract information at different levels and perform fusion for high-quality image reconstruction. In 2022, CSWin was proposed to reduce computational cost and overcome the limitations of local attention by computing self-attention only within the cross-shaped window [30,31,32]. This selective attention strategy is akin to the brain’s ability to focus on specific pathways for optimized information processing. The application of LePE addresses the limitation of self-attention where the width of the cross-shaped window based on network hierarchy updates limits the computational cost.

In the self-attention algorithm, the Query (*Q*), Key (*K*), and Value (*V*) matrices can also be considered as optimization objectives for the network’s width and depth. To improve the efficiency of neural networks related to Transformers, we propose an optimization method, the “Double-Attention (DA)” method, specifically targeting the self-attention mechanism. The network performance is enhanced by increasing the total number of parameters through increasing the network width and shifting the image in advance. Recent advancements in human attention research have significantly deepened our understanding of how attention is allocated and regulated during complex tasks. For instance, Posner and Rothbart introduced the three-network model of attention, elucidating the roles of alerting, orienting, and executive control in attentional processes [33]. Similarly, Corbetta and Shulman identified brain regions involved in spatial orientation and goal-directed behavior, shedding light on the neural mechanisms underlying attention [34]. These studies highlight the selective nature of human attention, wherein individuals prioritize stimuli pertinent to the current task while suppressing irrelevant information. This selectivity mirrors the weight allocation process in self-attention mechanisms, where the relevance of each input element is dynamically assessed to capture global dependencies effectively. Notably, self-attention mechanisms in neural networks have been inspired by these human attention processes, enabling models to focus on the most task-relevant parts of the input signal for further processing [35]. Moreover, recent research has explored the integration of human attention patterns into deep learning models to enhance their interpretability and performance. For example, researchers provided a comprehensive overview of efforts to incorporate human attention mechanisms into contemporary deep learning models, discussing future research directions and challenges [36]. Additionally, some researchers demonstrated that self-attention-based contextual modulation could improve neural response predictions, indicating that self-attention can enhance the model’s representational ability by refining the estimation of contextual information [37]. In summary, both human attention and self-attention mechanisms in neural networks exhibit the ability to selectively focus on pertinent information while integrating contextual cues, thereby optimizing processing efficiency and task performance.

In this study, DA includes two Key matrices ($K_{1}$ and $K_{2}$) that separately engage in matrix multiplication with the *Q* matrix. In contrast to the *Q*, $K_{1}$, and *V* matrices, the $K_{2}$ matrix is derived from the image after the “shifted window” operation. To enhance the training efficiency, we incorporate the information from adjacent windows during the preliminary stages of the process. The $K_{2}$ matrix broadens the network’s width and offers a supplemental set of evaluative criteria for pixel correlations between the *Q* and *V* matrices. This layered attention mechanism mirrors the hierarchical processing in human vision, where global context and fine details are simultaneously assessed.

The contributions of this work are summarized as follows:

- We propose a DA method introducing an additional Key matrix, generated from the image after the window-shifting operation, into the self-attention structure. This approach increases the width of the Swin Transformer network and the number of parameters, thereby enhancing the final accuracy. The additional Key matrix mimics the brain’s ability to incorporate auxiliary sensory inputs for refined decision-making.
- The DA method is applied to a dataset of kidney images from diabetic patients, provided by a medical institution. The reliability and feasibility of DA are validated through a biologically inspired integration of local and global attention mechanisms to achieve optimal balance in medical image analysis.

## 2. Methods

### 2.1. Similarities Between Human Attention and Self-Attention Mechanism

Human attention is a fundamental cognitive process that enables selective focus on relevant stimuli while suppressing irrelevant information. Similarly, the self-attention mechanism in neural networks assigns different levels of importance to various elements in input data, dynamically focusing on the most relevant parts. This section explores the parallels between these two systems, emphasizing their shared principles and functionalities.

#### 2.1.1. Selectivity and Relevance

Human attention is inherently selective, prioritizing information that is contextually or task relevant [33,34]. For example, in a cluttered environment, the human visual system focuses on objects of interest while ignoring background noise. Likewise, the self-attention mechanism uses Query and Key matrices to compute the relevance (or correlation) between different elements of input data. This relevance is quantified through a dot product operation, followed by a softmax function to assign weights, enabling the model to focus on the most pertinent information [2].

#### 2.1.2. Contextual Integration

Human attention integrates information from the surrounding environment to form a coherent understanding. This process involves dynamic shifts in focus, such as shifting gaze between regions of a scene. The self-attention mechanism mirrors this ability by calculating correlations between all elements of the input data, capturing global dependencies. The inclusion of additional Key matrices in the DA method extends this concept by explicitly modeling interactions between adjacent regions, similar to how humans synthesize local and global information.

#### 2.1.3. Dynamic Resource Allocation

In human cognition, attention dynamically allocates neural resources based on task demands, ensuring efficient processing [33]. Similarly, self-attention mechanisms adjust the weights of elements in the input data, emphasizing critical features while de-emphasizing redundant or irrelevant ones. This dynamic allocation optimizes computational resources, much like the human brain optimizes cognitive effort.

#### 2.1.4. Feedback and Adaptation

The human attention system adapts based on feedback, such as when learning from mistakes or adjusting focus during task execution. In neural networks, this adaptability is reflected in the backpropagation process, where the weights of the Query, Key, and Value matrices are updated to improve performance. The introduction of an additional Key matrix in the DA mechanism enhances this adaptability by allowing more nuanced adjustments during training.

#### 2.1.5. Multi-Task Capabilities

Human attention enables multi-tasking by distributing focus across different stimuli. The multi-head self-attention mechanism in Transformers embodies this principle by using multiple attention heads to learn diverse features from input data, enhancing model generalization and performance.

### 2.2. Overall Architecture

**Figure 1:**
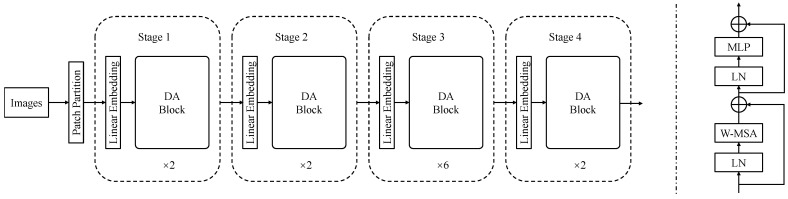
The architecture of the DA method in the Transformer.

The overall structure of the DA Transformer is shown in Figure 1, which was constructed based on the Swin Transformer with a window size of 7. The input image is firstly divided into non-overlapping patches through the Patch Partition Block. Then, the image shape is modified by the Embedding layer before being fed into the DA Transformer Block for self-attention computation. In Stage 2, Stage 3, and Stage 4, the feature maps are downsampled using the Patch-Merging layer and then input into the DA Transformer Block.

The DA Transformer Block, as shown on the right side of Figure 1, takes the feature map as input and passes it through a LayerNorm (LN) layer. The output from the LN layer is then fed into the Window-Based Multi-Head Self-Attention (W-MSA) layer. After applying residual connections, the output passes through another LN layer and then into the Multi-Layer Perceptron (MLP) layer. Similar to the Swin Transformer, residual connections are applied after the MLP layer. However, different from the Swin Transformer, the shifted-window operation is not applied after the first usage. This means that the second Key matrix is indeed involved in the network.

The introduction of an additional matrix $K_{2}$ in the DA method significantly enhances the model’s ability to capture cross-pixel relationships, offering both theoretical and practical advancements. By leveraging $K_{2}$, derived from shifted images, the DA mechanism explicitly encodes inter-window correlations, integrating global context while eliminating the redundant computations found in traditional self-attention methods. This design expands the network’s capacity without a proportional increase in computational complexity, contrasting with methods like the Swin Transformer, which rely on shifted-window operations with additional overhead. The DA mechanism aligns with human attention systems, where contextual integration across regions optimizes perception. This biologically inspired approach reduces processing time, accelerates training convergence, and maintains high accuracy, demonstrating its suitability for large-scale datasets and resource-limited scenarios. Its theoretical innovation and practical utility highlight a unique contribution to the design of Transformer-based models, offering a streamlined yet powerful alternative to mainstream approaches.

### 2.3. Self-Attention with Another Key Matrix

**Figure 2:**
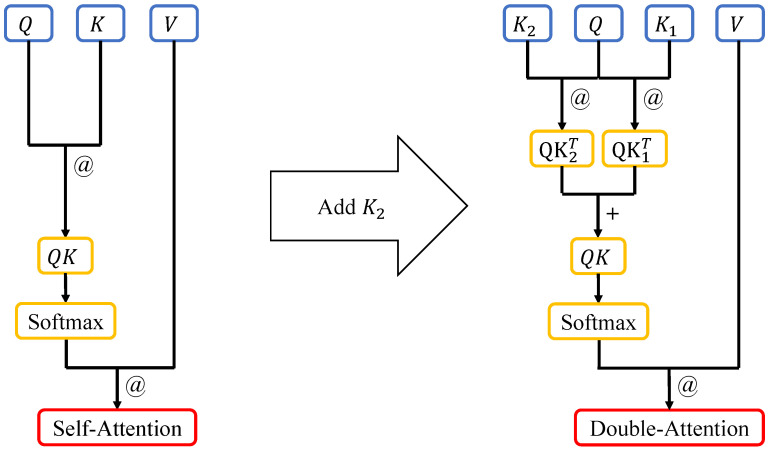
Self-attention algorithm schematic diagram (**left**) and Double-Attention algorithm schematic diagram (**right**). The $K_{2}$ matrix is multiplied by the *Q* matrix, and the resulting matrix is added to the computation result of the $K_{1}$ matrix. After applying the softmax function, the resulting values are multiplied by the *V* matrix.

In Figure 2, on the left side is the flowchart of the self-attention algorithm. The process reflects an abstraction of how human attention dynamically adjusts focus based on the relevance of input information. The dot product (Mark: “@”) of the Query matrix and the Key matrix is performed, and then the softmax values are calculated row-wise. Then, the dot product of the resulting value and the Value matrix are taken. On the right side is the flowchart of our improved self-attention algorithm. Inspired by human selective attention mechanisms, first, the dot product of the Query matrix and the $K_{1}$ and $K_{2}$ matrices is taken. Then, the results are added (Mark: “+”) element-wise, and softmax values are calculated row-wise. Finally, the dot product of the softmax values and the Value matrix is taken.

The dot product (@) is a well-known method for computing the similarity between two vectors along the same direction [38,39]. In the context of self-attention, it resembles the evaluation of perceptual similarity in human cognition, where certain patterns or features are matched to allocate focus. When it is applied in the matrix computation of self-attention, it can be seen as calculating the similarity between matrices, specifically, the correlation between their elements. The Query and Key matrices are used to compute the correlation between them, while the Value matrix represents the information extracted from the input data [2,40]. The process of taking the dot product between the softmax probabilities and the input information, after softmax calculation, determines the importance of the input information. This mechanism aligns with the way human attention weighs sensory inputs, prioritizing relevant stimuli while suppressing distractions.

In the DA method, a new Key matrix is introduced, which increases the overall width of the neural network and provides more parameters. This adjustment reflects the redundancy in human neural processing, where multiple pathways often contribute to enhanced perception and decision-making. The presence of an additional matrix implies that the *Q* matrix can be adjusted more significantly during the backpropagation process, allowing the model to achieve the target accuracy earlier than models with only one Key matrix. This mirrors how human attention mechanisms adapt dynamically to improve task performance by refining focus through feedback.

In the self-attention mechanism, the gradients for the *Q*, *K*, and *V* matrices are derived from the loss function *L*. The gradients of the output *Z* with respect to the inputs can be computed as follows:

(1) $$\frac{\partial L}{\partial Q},\frac{\partial L}{\partial K},\frac{\partial L}{\partial V}.$$

For a standard self-attention process, the gradient’s primarily flow through the attention scores is as follows:

(2) $$A=softmax(\frac{QK^{T}}{\sqrt{d_{k}}}),$$

where each element of *A* represents the normalized correlation between *Q* and *K*. In the DA mechanism, the attention score can be modified as follows:

(3) $$A_{DA}=softmax\left(\frac{QK_{1}^{T}}{\sqrt{d_{k}}}+\frac{QK_{2}^{T}}{\sqrt{d_{k}}}\right).$$

This addition introduces new pathways for gradient propagation through $K_{2}$. The gradient of $K_{1}$, $K_{2}$, and *Q* can be written as follows:

(4) $$\frac{\partial L}{\partial K_{1}}=\frac{\partial L}{\partial A_{DA}}\frac{\partial A_{DA}}{\partial K_{1}},\frac{\partial L}{\partial K_{2}}=\frac{\partial L}{\partial A_{DA}}\frac{\partial A_{DA}}{\partial K_{2}},\frac{\partial L}{\partial Q}=\frac{\partial L}{\partial A_{DA}}\left(\frac{\partial A_{DA}}{\partial K_{1}}+\frac{\partial A_{DA}}{\partial K_{2}}\right).$$

The inclusion of $K_{2}$ introduces an additional gradient path, effectively diversifying the optimization process. This redundancy in the gradient flow stabilizes training by mitigating vanishing or exploding gradients in certain regions. During backpropagation, the parameters of $K_{1}$, $K_{2}$, and *Q* are updated according to their respective gradients:

(5) $$K_{1}^{\left(t+1\right)}=K_{1}^{\left(t\right)}-\eta \frac{\partial L}{\partial K_{1}},K_{2}^{\left(t+1\right)}=K_{2}^{\left(t\right)}-\eta \frac{\partial L}{\partial K_{2}},Q^{\left(t+1\right)}=Q^{\left(t\right)}-\eta \frac{\partial L}{\partial Q},$$

where η is the learning rate.

The additional gradient flow through $K_{2}$ allows for more nuanced updates of *Q* during training, leading to faster convergence and enhanced accuracy. This property is particularly advantageous in scenarios with complex data dependencies, where traditional self-attention mechanisms may struggle to efficiently propagate gradients.

### 2.4. Key Matrix Generated by Shifted Image

In the self-attention algorithm, the Query, Key, and Value matrices are all computed from the input data through the Embedding layer or Patch-Merging layer. This process is analogous to how the human brain extracts features from raw sensory inputs, such as identifying edges, shapes, or patterns in a visual scene. Therefore, the results obtained through the Transformer layer represent the importance of the input data. Similar to human attention mechanisms, the computed importance reflects a prioritization of certain features that are most relevant to the task at hand.

**Figure 3:**
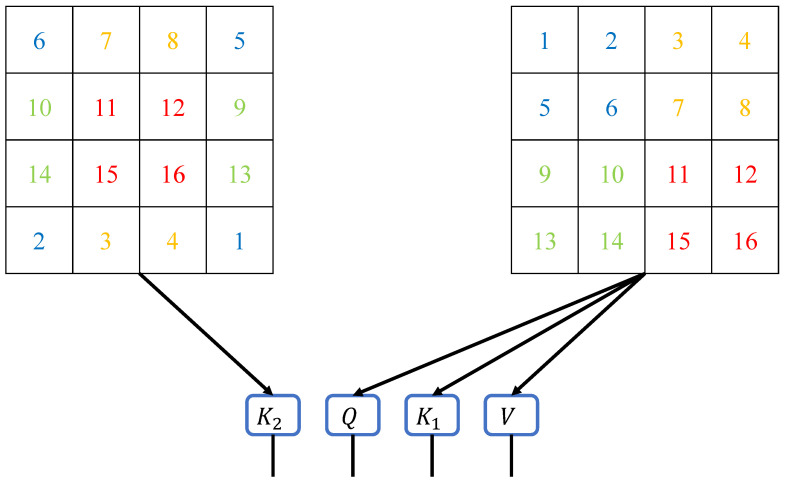
Matrix generation schematic diagram for the DA method. The matrices *Q*, $K_{1}$, and *V* are generated from the original input image, while the $K_{2}$ matrix is generated from the shifted input image. The multiplication of the $K_{2}$ matrix with the *Q* matrix can be seen as the interaction between different windows, which resembles the process of shifting and recalculating windows in the Swin Transformer.

As shown in Figure 3, we generate the $K_{2}$ matrix from the shifted image. This operation is performed synchronously with the generation of the Query, Key, and Value matrices. By introducing an additional Key matrix derived from spatially shifted data, our method mirrors how human attention integrates context from adjacent areas to form a cohesive understanding of the environment. In this manner, the correlations between different windows already exist when calculating the DA, eliminating the need to compute self-attention again. This approach is inspired by the human ability to anticipate relationships between different regions of focus, enabling efficient processing without redundant computations. Therefore, the shifted-window component is adjusted to effectively reduce the training time of the neural network. This adjustment reflects the efficiency of human attention systems, which optimize resource allocation by avoiding unnecessary repetitive processing.

### 2.5. Comparison of Computational Complexity

As shown in Figure 2, the self-attention mechanism applied in the Swin Transformer involves two matrix multiplication operations and one softmax calculation. In the DA method, there are three matrix multiplication operations, one matrix addition operation, and one softmax calculation. However, as shown in Figure 1, the DA method utilizes the same number of Transformer blocks as the Swin Transformer but the amount of computation is slightly higher than that of the Swin Transformer because the newly added matrix brings more parameters. However, due to the limitation of the network structure on the computational scale, the increased amount of computation is still within an acceptable range.

The comparison equations for the calculation of the amount of multi-head self-attention (MSA) and W-MSA are as follows [6]:

(6) $$\begin{array}{cc} \; & \Omega \left(\mathrm{MSA}\right)=4hwC^{2}+2{\left(hw\right)}^{2}C, \end{array}$$

(7) $$\begin{array}{cc} \; & \Omega \left(W\text{-}\mathrm{MSA}\right)=4hwC^{2}+2M^{2}hwC, \end{array}$$

Meanwhile, *w* and *h* represent the width and height of the input data, respectively. *C* denotes the depth of the input data, while *M* indicates the size of each window. The DA method extends the W-MSA by incorporating the $K_{2}$ matrix. This extension entails additional steps, such as generating the $K_{2}$ matrix and multiplying the *Q* matrix by the $K_{2}$ matrix. According to Equation (7), the computational complexity of DA is as follows:

(8) $$\begin{array}{c} \Omega \left(\mathrm{DA}\right)=5hwC^{2}+3M^{2}hwC. \end{array}$$

Among the related methods, the Medical Transformer is a neural network with low computational resource requirements in the field of medical images [25]. The computational complexity of gated axial-attention (GAA) can be expressed as follows:

(9) $$\begin{array}{c} \Omega \left(\mathrm{GAA}\right)=4hwC^{2}+2\left(h^{2}+w^{2}\right)C, \end{array}$$

where the part of the matrices’ computational complexity has been optimized. Comparing Equations (8) and (9), it is challenging to definitively determine which method exhibits lower computational complexity. However, samples in medical image datasets often possess high resolution and are extremely abundant. This will increase the computational complexity of the GAA method. Consequently, the DA method can still be effectively trained with fewer parameters.

## 3. Results

### 3.1. Experimental Setup

In this section, publicly available pre-trained files are applied as the initial data for each experiment. Considering DA involves changes by the number of parameters in the self-attention algorithm, the pre-trained files cannot be directly employed. Therefore, all parameters related to the self-attention algorithm from the pre-trained files are removed. This modification allows for a direct and intuitive comparison of the results. On each benchmark dataset, the following algorithms are compared: W-MSA, DA-N, DA, and ViT. W-MSA represents the self-attention mechanism without modifications. DA-N denotes the method of generating the $K_{2}$ matrix from the original image based on W-MSA. DA represents the method of generating the $K_{2}$ matrix from the shifted image based on W-MSA. ViT is the Vision Transformer method, applied for comparison in terms of the number of parameters and FLOPs.

In order to verify the versatility of our methods, we also make the same improvements in the Cswin and SimMIM methods. The Cswin Transformer(Cswin) [30], SimMIM [41], and Astroformer [24] are also improved by DA. In the experiments, the configurations of W-MSA, DA-N, and DA all belong to “Tiny” in the Swin Transformer. The configuration of ViT is “Base”. The configurations of Cswin, SimMIM, and Astroformer are “Small”, “Base”, and “Tiny”.

This paper’s research utilizes five publicly available datasets to ensure experiment reproducibility and data transparency. The datasets used in this study are CUB-200 [42], Oxford-IIIT Pet [43], Flower-102 [44], Food-101 [45], CIFAR-100 [46], and Tiny ImageNet [47]. All of these datasets are openly accessible, and researchers can find detailed information as well as download links on the official websites or in the relevant papers.

To prevent experimental data from being reused, the datasets are separated in advance according to the initial state of the benchmark datasets. To ensure the objectivity of the experimental results, the data of the training set are more divided. For instance, for the Oxford-IIIT Pet dataset, the authors divide it into a training set and a test set. The data of the test set remain unchanged, and part of the data of the training set is separated as a validation set.

The results of the experiment are presented in the form of tables and line graphs. The results in the tables are the accuracy on the test set, that is, the model with the best performance on the validation set is selected during the training process, and then the test set is run on this model. The line graph shows the trend of accuracy on the training and validation sets throughout the training process. That trend can be used to visualize the efficiency of different methods as well as the gap.

### 3.2. Experiments on Benchmark Datasets

The results of the comparative experiments on the CUB-200 dataset are presented in Table 1. As shown in the table, the accuracy achieved by the DA-N method is higher than that of the W-MSA method, demonstrating that incorporating the $K_{2}$ matrix can improve accuracy by increasing the network’s width. The DA method yields higher accuracy than DA-N, indicating that the $K_{2}$ matrix generated from the shifted image effectively facilitates information interaction within the network.

Furthermore, the number of parameters and FLOPs for the DA method is significantly lower than for the ViT.

**Figure 4:**
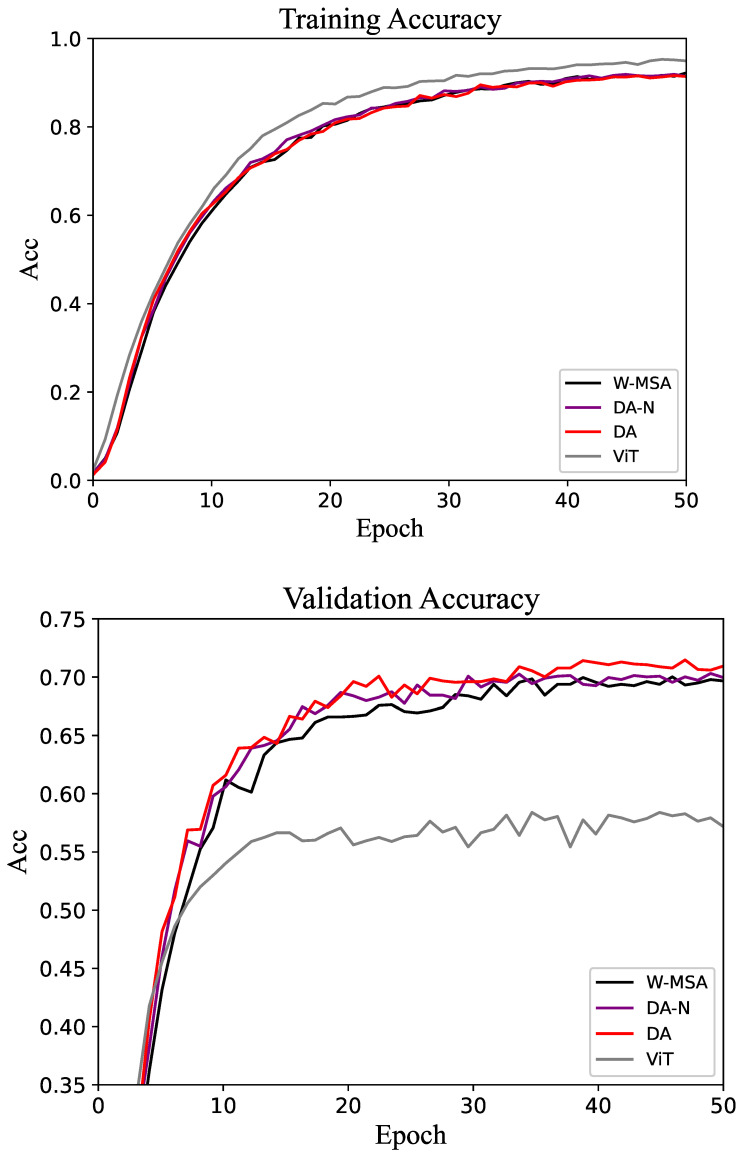
Accuracy of the training and validation sets in the CUB-200 dataset.

For the CUB-200 dataset, the accuracy of the training and validation sets is shown in Figure 4. As the epoch increases, the accuracy of the model gradually stabilizes. On the validation set, the accuracy of DA tends to be higher than that of W-MSA and DA-N. The performance proves that the DA method is more efficient than the control groups.

**Figure 5:**
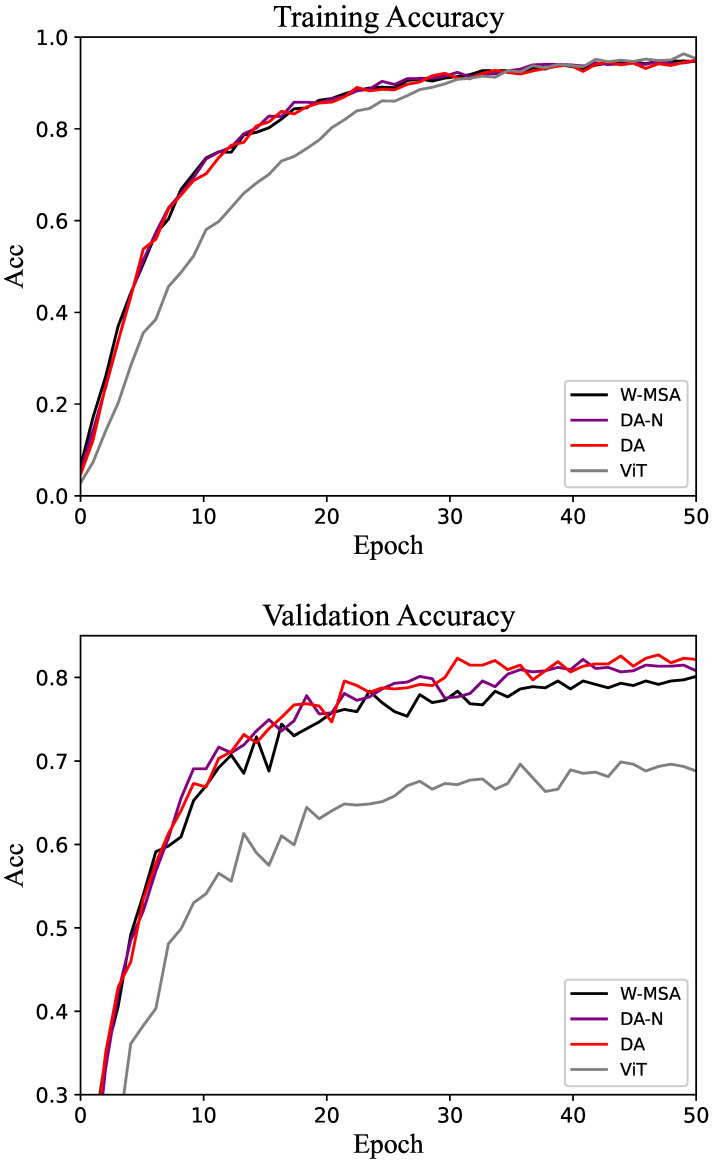
Accuracy of the training and validation sets in the Oxford-IIIT Pet dataset.

The experimental results for the Oxford-IIIT Pet dataset are shown in Table 2. The overall trend of the experimental results is consistent with those observed for the CUB-200 dataset, with the DA method achieving the highest accuracy. As shown in Figure 5, both on the training set and validation set, the DA method has an obviously better performance than the control groups.

**Figure 6:**
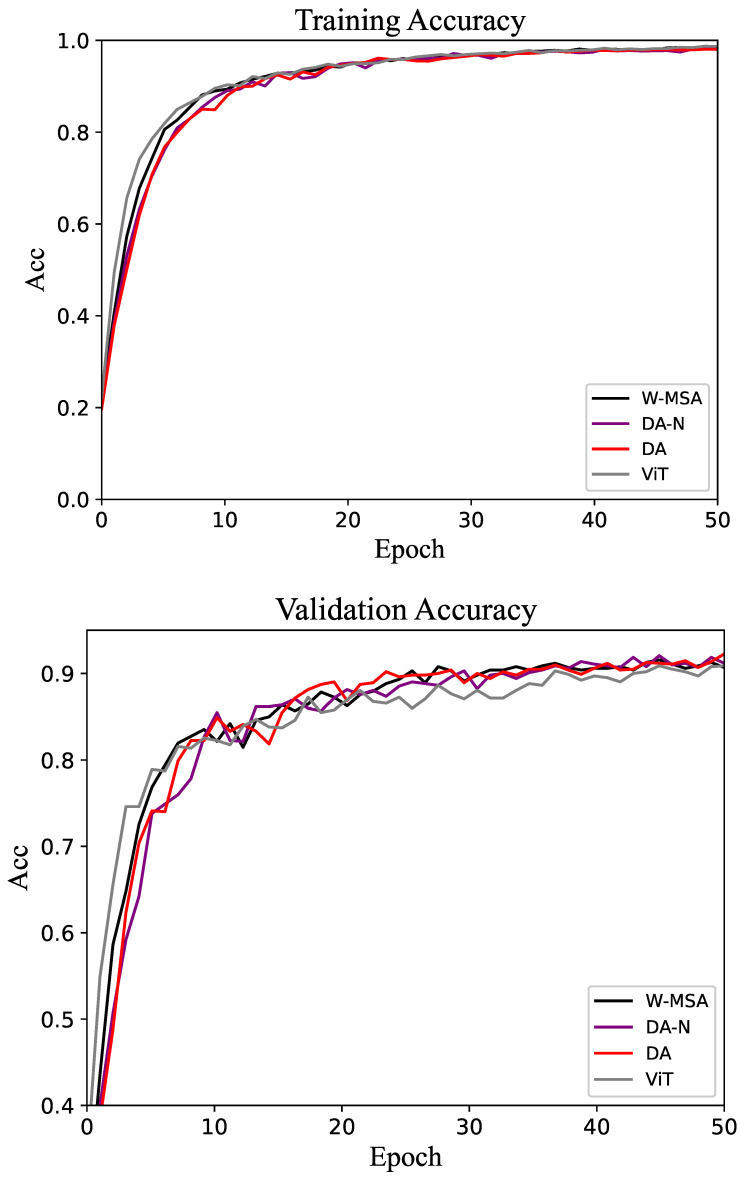
Accuracy of the training and validation sets in the Flower-102 dataset.

**Figure 7:**
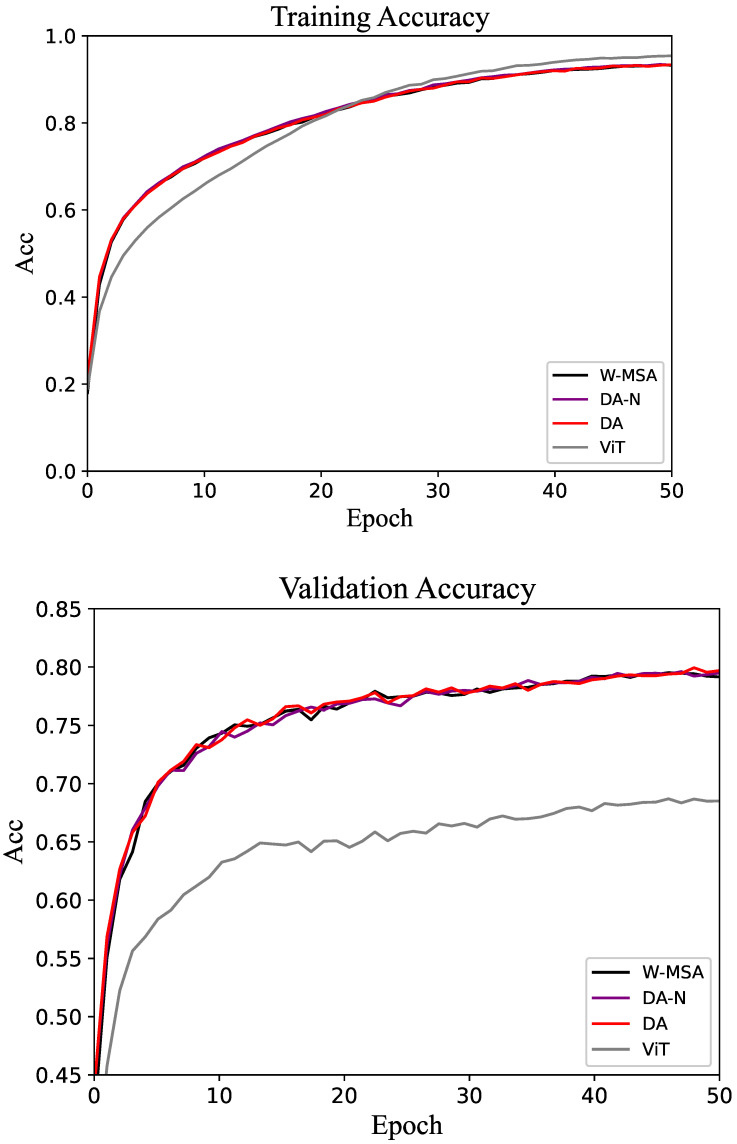
Accuracy of the training and validation sets in the Food-101 dataset.

The comparative experimental results for the Flower-102 and Food-101 datasets are shown in Table 3 and Table 4, respectively. The accuracy change curves of these two datasets are shown in Figure 6 and Figure 7. As expected, the proposed method outperforms the baseline in both cases. Different from the results shown in Table 1 and Table 2, all methods achieve relatively high accuracy on these two datasets. This is because, with sufficiently high image resolution, the entire network can be fully trained within a limited number of epochs. In the validation set of the Flower-102 and Food-101 datasets, the accuracy of the DA method is often above that of the control groups.

**Figure 8:**
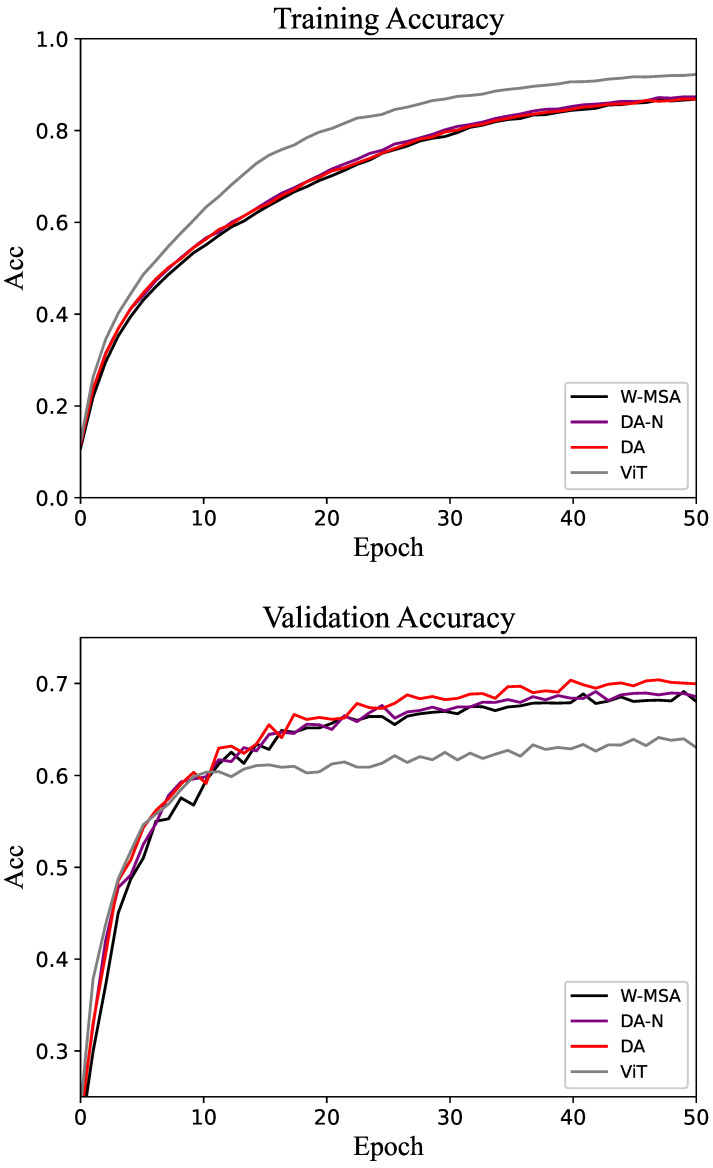
Accuracy of the training and validation sets in the CIFAR-100 dataset.

The comparative experimental results for the CIFAR-100 dataset are shown in Table 5. The accuracy change curves of the datasets are shown in Figure 8. Although the proposed method still outperforms the baseline, the overall accuracy is relatively low. This is primarily due to the small size of the CIFAR dataset samples, which poses challenges for achieving higher accuracy. In the validation set, the accuracy of the DA method is above that of the control groups at most epochs.

The comparative experimental results for the Tiny ImageNet dataset are shown in Table 6. The accuracy of the DA method is also higher than that of other methods. This proves that the DA method still has a better performance.

The comparative experimental results for the Flower-102 dataset with Cswin, SimMIM, and Astroformer are shown in Table 7, Table 8 and Table 9. The final accuracy of the DA method is above that of the control groups at most epochs. This proves that DA and the methods both have good versatility in Swin-derived methods.

Experiments on various datasets provide strong evidence for the effectiveness of the DA method. The accuracy is effectively improved by generating $K_{2}$ matrices from the shifted image.

### 3.3. Experiments on Medical Dataset

**Figure 9:**
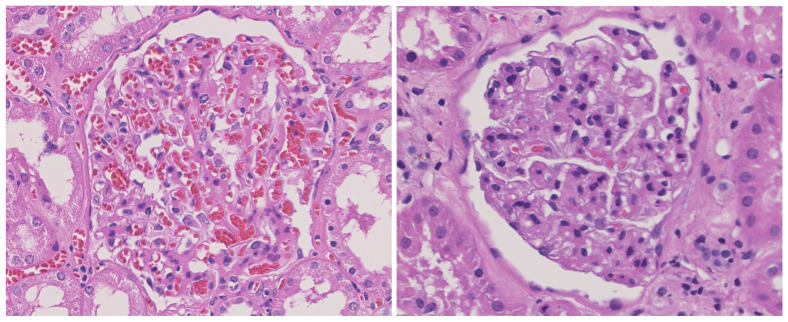
Example sample of kidney imaging dataset.

In order to evaluate the performance of the proposed method in real-world applications, the experiments were conducted on a kidney imaging dataset of diabetic patients. Example samples from this dataset are illustrated in Figure 9. The left figure depicts a kidney affected by diabetes, and the right figure shows a non-diabetic control kidney. Note that the sick subset contains pictures of people with mild diabetes. Such photos are not easy to distinguish even by doctors. Therefore, the judgment result of the DA in this study can provide effective assistance for doctors or researchers. The dataset consists of 14,249 data samples, with 5952 samples representing kidneys affected by diabetes and 8297 samples representing healthy kidneys. The dataset was divided into train, val, and test subsets. The preprocessing of data samples included rotation and flipping, etc. The sub-datasets were divided according to the most commonly used 6:2:2 ratio [48,49].

The experimental results for the kidney imaging dataset are presented in Table 10. The DA method achieves higher accuracy compared to W-MSA and DA-N. It is worth noting that the samples in the dataset have larger dimensions, typically around 2000 × 2000, which is larger than most benchmark datasets. High-resolution images and sufficient sample numbers allow the network to be adequately trained within a limited number of epochs. Therefore, in medical applications, even if the accuracy of the DA method is slightly lower than that of ViT, its advantage in computational efficiency is sufficient to compensate for the minor 0.3 percent accuracy gap.

## 4. Discussion and Conclusions

The proposed Double-Attention (DA) mechanism addresses computational efficiency and accuracy challenges in Transformer-based neural networks. By introducing an additional Key matrix ($K_{2}$) derived from shifted images, the DA mechanism expands the network width and enhances its capacity to capture inter-pixel relationships across different windows. This approach aligns with previous studies on optimizing self-attention mechanisms but eliminates redundant computations, resulting in faster training.

Our results indicate that the inclusion of $K_{2}$ improves the model’s ability to learn feature representations, thereby accelerating convergence during training. This finding supports previous studies suggesting that redundancy and modularity in neural networks, akin to biological attention systems, can enhance learning and generalization capabilities. Moreover, the removal of the shifted-window component highlights the efficiency gains achieved by embedding cross-window correlations directly within the self-attention calculation.

From a theoretical perspective, this method exemplifies how human attention mechanisms can inspire computational models. The integration of additional contextual information, as demonstrated by $K_{2}$, mirrors the brain’s capacity to process information from adjacent regions to form cohesive perceptions. This biologically inspired design principle contributes to the ongoing dialogue on how neural networks can emulate human cognitive processes to achieve more efficient computation.

Future research could explore integrating the DA method into multi-modal tasks, such as combining visual and textual data for applications like image–text retrieval or segmentation tasks. By introducing modality-specific Key matrices, DA could enhance cross-modal feature alignment and representation learning, making it a versatile tool for multi-modal systems.

## Figures and Tables

**Table 1:** Comparison of different methods applied to CUB-200 classification.

Method	Acc (%)	FLOPs (G)	#param. (M)
W-MSA	69.4	4.4	27.7
DA-N	70.6	4.7	29.8
**DA**	72.6	4.7	29.8
ViT	58.9	16.9	85.8

**Table 2:** Comparison of different methods applied to Oxford-IIIT Pet classification.

Method	Acc(%)	FLOPs(G)	#param. (M)
W-MSA	69.3	4.4	27.5
DA-N	73.6	4.7	29.7
**DA**	74.2	4.7	29.7
ViT	58.2	16.9	85.7

**Table 3:** Comparison of different methods applied to Flower-102 classification.

Method	Acc (%)	FLOPs (G)	#param. (M)
W-MSA	90.7	4.4	27.6
DA-N	91.8	4.7	29.7
**DA**	92.4	4.7	29.7
ViT	89.2	16.9	85.7

**Table 4:** Comparison of different methods applied to Food-101 classification.

Method	Acc (%)	FLOPs (G)	#param. (M)
W-MSA	84.3	4.4	27.6
DA-N	84.4	4.7	29.7
**DA**	84.5	4.7	29.7
ViT	74.5	16.9	85.7

**Table 5:** Comparison of different methods applied to CIFAR-100 classification.

Method	Acc (%)	FLOPs (G)	#param. (M)
W-MSA	67.8	4.4	27.6
DA-N	69.4	4.7	29.7
**DA**	70.2	4.7	29.7
ViT	63.2	16.9	85.7

**Table 6:** Comparison of different methods applied to Tiny ImageNet dataset.

Method	Acc (%)	FLOPs (G)	#param. (M)
W-MSA	60.7	4.4	27.6
DA-N	60.5	4.7	29.8
**DA**	61.6	4.7	29.8
ViT	52.1	16.9	85.8

**Table 7:** Comparison of different Cswin-like methods applied to Flower-102 classification.

Method	Acc (%)	FLOPs (G)	#param. (M)
W-MSA	69.3	6.4	34.2
DA-N	70.1	6.9	36.9
**DA**	70.5	6.9	36.9

**Table 8:** Comparison of different SimMIM-like methods applied to Flower-102 classification.

Method	Acc(%)	FLOPs (G)	#param. (M)
W-MSA	71.6	15.2	86.8
DA-N	72.8	16.4	93.8
**DA**	74.8	16.4	93.8

**Table 9:** Comparison of different Astroformer-like methods applied to Flower-102 classification.

Method	Acc (%)	FLOPs (G)	#param. (M)
W-MSA	90.5	4.4	27.6
DA-N	91.2	4.7	29.7
**DA**	91.8	4.7	29.7

**Table 10:** Comparison of different methods applied to diabetes classification.

Method	Acc (%)	FLOPs (G)	#param. (M)
W-MSA	91.6	4.4	27.5
DA-N	93.2	4.7	29.7
**DA**	96.6	4.7	29.7
ViT	96.9	16.9	85.7

## Data Availability

The data used in this study are limited access but available for reasonable requirements. Interested parties may request access by contacting the corresponding author at yktodo@se.kanazawa-u.ac.jp. Access is subject to approval and compliance with confidentiality and ethical guidelines. The authors are committed to facilitating access within legal and ethical boundaries.
